# Pharmacogenomics based precision medicine in gastroesophageal cancers: way to move forward?

**DOI:** 10.18632/oncoscience.520

**Published:** 2020-09-09

**Authors:** Shrunjal Shah, Sarbajit Mukherjee

**Affiliations:** ^1^Department of Medicine, Roswell Park Comprehensive Cancer Center, University at Buffalo School of Medicine, Buffalo, NY, USA

**Keywords:** gastric cancer, esophageal cancer, oxaliplatin, pralatrexate, single nucleotide polymorphisms (SNPs)

Gastroesophageal cancers (GEC), combined, are the most prevalent type of gastrointestinal cancers worldwide. Esophageal cancer is a global health problem with an estimated annual incidence of 455,800 and 400,200 deaths occurring worldwide in 2012 [1]. On the other hand, gastric cancer is responsible for approximately 952,000 new diagnoses globally (6.8% of new cancer cases worldwide) and 723,000 deaths annually (8.8% of total) [2]. Patients with metastatic or surgically inoperable GEC have a somber prognosis, with a median overall survival of 10-12 months, and palliative chemotherapy is the mainstay of treatment [3]. New treatments like HER2 directed therapy, vascular endothelial growth factor receptor inhibitors and immune checkpoint inhibitors have demonstrated improvements in survival. However, despite these latest advances, the prognosis of patients with advanced GEC remains dismal, highlighting the need for novel drug combinations.

We performed a phase II clinical trial of oxaliplatin combined with pralatrexate in metastatic or surgically inoperable, treatment naïve, GEC patients with a primary objective to assess the overall response rate (ORR). Our secondary objectives included the toxicity and tolerability of this regimen, time to progression, and overall survival. We also aimed to investigate whether functionally relevant polymorphisms of genes in the folate metabolism pathway were associated with the toxicity and efficacy of pralatrexate. As a correlative study, we wanted to assess whether microRNA expression profiling of the tumor's epithelial component can be used as a predictive biomarker to treatment response [4].

In this study, a total of 35 patients were enrolled Four patients were treated at an initial dose level of Oxaliplatin 85 mg/m2 combined with pralatrexate 120 mg/m2, and two patients developed dose-limiting toxicities. Pralatrexate was dose reduced to 100 mg/m2 for the rest of the subjects (N=31). The median age of the overall population was 67 years, and adenocarcinoma was the predominant histology (83%). An ORR of 26% (95% CI, 12–43) was observed in 35 patients. The study did not achieve the defined primary endpoint of efficacy. The progression-free survival (PFS) was estimated at 5.1 (95% CI 3.4-6.4) months, and overall survival (OS) was 7.2 ( 95% CI 6.4-10.8) months. As for the toxicity profile of pralatrexate, the most common grade 3 or higher events were mucositis, neutropenia, and dehydration. Overall, the toxicities were manageable, and the therapy was well tolerated with the reduced pralatrexate dose.

Pralatrexate differs from other antifolates, both structurally and mechanistically, which leads to differences in antitumor activity [5]. It selectively enters cells expressing reduced folate carrier (RFC-1), encoded by the gene SLC19A1. The structure of pralatrexate allows enhanced intracellular drug retention and the formation of polyglutamylated metabolites compared to other antifolates. Given that pralatrexate has demonstrated synergy with platinum and taxane-based compounds, this study looked at the combined efficacy of pralatrexate and oxaliplatin.

Studies have shown that loss of function mutations in the SLC19A1 gene make cancer cell lines resistant to antifolates in vitro [6]. Several polymorphisms have been identified for SLC transporter genes, but their functional relevance concerning drug response and clinical outcomes remains unclear [7]. Interestingly, in this study, the SLC19A1 rs28389757 polymorphisms were associated with an improved PFS and rs1051255 with improved OS. Similarly, specific polymorphisms in DHFR and GGH genes were associated with survival. Pharmacogenomics is evolving rapidly due to emerging technologies and increasing knowledge of pathways of drug metabolism. It is becoming more apparent that a personalized, genotype-based chemotherapy dose might be feasible and less toxic in GEC patients [8].

Conventional serum biomarkers, such as CEA, are not useful in GEC. This study examined whether microRNA (miRNA)s can be used as a potential biomarker. MicroRNAs regulate cell proliferation, differentiation, and apoptosis, and depending on its mRNA target; a miRNA can function as a tumor suppressor or promoter of oncogenesis [9]. Besides, miRNAs are likely to predict response to chemotherapy [10]. We used different methods like principal component analysis, unsupervised hierarchical clustering, and differential expression analyses, however, an association between tumor epithelial microRNA expression and disease progression could not be identified. Future studies should address the prognostic and predictive values of miRNAs in larger, independent datasets.

In conclusion, the treatment of advanced GEC remains a significant challenge, and many questions remain unresolved. There is an unmet need for intensive research to identify biomarkers of both predictive and prognostic significance. In this new era of pharmacogenomics research and precision medicine, the future is exciting.
